# Toward More Inclusive Work Organizations by Redesigning Work

**DOI:** 10.3389/fresc.2022.861561

**Published:** 2022-05-31

**Authors:** Henny Mulders, Gemma van Ruitenbeek, Bert Wagener, Fred Zijlstra

**Affiliations:** ^1^Dutch Employee Insurance Agency (UWV), Amsterdam, Netherlands; ^2^Department of Work and Social Psychology, Faculty of Psychology and Neuroscience, Maastricht University, Maastricht, Netherlands; ^3^University of Applied Sciences of the German Social Accident Insurance (HGU), Bad Hersfeld, Germany

**Keywords:** inclusion, work redesign, rehabilitation, disability, vulnerable labor market groups, PES, ALMP, SHRM

## Abstract

Inclusive Work Redesign (IWR) is an innovative strategy to create feasible job opportunities for job seekers from vulnerable groups at the labor market, in particular people with disabilities, in such a way that it contributes to sustainable employment for all staff and to the organization's performance. As an element of active labor market policies (ALMP), in the Netherlands, IWR is used as an instrument for Public Employment Services (PES) and professionals in occupational rehabilitation to support employers, using a demand-oriented approach. IWR is also a potential valuable asset for strategic human resource management (SHRM) aimed at inclusion and sustainable employment. The article describes IWR in the context of social security and staffing problems of organizations, and the application of IWR in the Netherlands and Germany.

## Introduction

Including people with disabilities into the world of work is still a great challenge for work organizations all over the world. Nevertheless, organizations should seriously consider all options to become more inclusive, for their own sake and that of society at large. People with disabilities often do not fit into regular jobs that are becoming too demanding even for an increasing number of workers without disabilities. In order to promote sustainable employability for all people, both workers and job seekers, there is a need for innovative work redesign, aimed at a better fit of work to individual abilities and ambitions.

Efforts of occupational rehabilitation professionals to (re)integrate people with disabilities into regular jobs still are predominantly “supply-oriented”. They are supporting people individually in their search for a suitable job, their integration or return-to-work. These efforts are in need of a complementary “demand-oriented” approach, supporting employers to create suitable jobs (1, 2). For this purpose, the method “Inclusive Work Redesign” (IWR) was developed^1^ in the Netherlands. During the past decade, this approach has been applied within the context of social security in “inclusive consulting” a great number of Dutch organizations (both public and private) in many different branches. Supported by the European Social Fund, researchers and social security practitioners from Germany and the Netherlands recently started a transnational co-operation to explore the practical suitability of IWR in the German context.

## The Rise of Active Labor Market Policies

Starting in the 1980s, governments in many countries with a developed social security system adopted new policies in reaction to a steadily growing number of people dependent on allowances and as an essential element of austerity. They introduced all kinds of measures in order to limit expenses and encourage jobless people to (re)enter the labor market, that became known as Active Labor Market Policies (ALMP) (3). Important components of ALMP are public employment services and public human capital investments [assessment, vocational guidance, education and training, and recently “up-skilling and re-skilling” (4)]. For people with disabilities, a “paradigm shift” was proclaimed by the OECD (5), “from compensation to participation”. This was followed by intensification of ALMPs for this particular group of people in member states (6). In fact, this led to a profound change in the nature of social security. From now on, the adage was social security by employment: anyone able to work, has to work and stay at work; allowances are only for fully incapacitated persons.

As a result, people with disabilities not only have the universally proclaimed *right* (7) to participate in work, but many of them nowadays also have an inevitable *duty* to work. This makes the need for suitable jobs for them even more urgent. This shift in social security also places the employers at the center of the social security system as the ones who are supposed to be able and willing to provide those suitable jobs. IWR was developed to support employers in pursuing this role.

### Work Organizations and Their New Role in Social Security

The vast majority of employers still turns out to be reluctant to hire people with disabilities (8, 9). There is a complex of different reasons behind this negative attitude that does not match their supposed new role in social security at all. Doubts about the employability of people with disabilities, supposed lower productivity, higher absence rates, and aversion of co-workers are amongst the most frequently documented reasons for not hiring them (10). Most prominent, however, is the argument that there are *no suitable jobs* in their organization for jobseekers with disabilities (11). Because this is a final argument, “it is not possible at all”, it is very important to take a closer look at the way work is organized nowadays.

Division of labor (functional differentiation, specialization) and coordination (integration of effort) are important determinants of a work organization, resulting in particular jobs, roles and responsibilities (12, 13). These actual work design features are the result of human decisions based on practical and theoretical considerations, under the strong influence of social, economic and technological developments and institutional context (12, 14) and long-standing evolved work (re)design traditions (15). Apparently, these features are unfavorable to people with disabilities as well as other jobseekers whose knowledge, skills and abilities (KSAs) do not match the qualifications asked for in the labor market.

### Opposite Trends, Toward More Exclusive Organizations

Profound changes in the nature of work over the past three decades had great consequences for people at work and job seekers. Cognitive and social skills are becoming predominant in future work. There is an increasing need for the *combination* of problem solving skills and interpersonal skills (16). Overall, work has become more and more demanding. Traditional branches of agriculture and industry had to give up their long-standing predominant position, in particular in favor of the public and private service sector and healthcare. Global competition and technological developments acted as main drivers for highly efficient workplaces. Quality of work and working conditions were not primary goals in this course of events, and were put under pressure (17, 18). On one hand, task complexity increased, tasks became more flexible (job rotation), more individual responsibility was needed (task autonomy) and there was a higher need for social skills in teamwork, in contact with customers in service work or with patients in healthcare. On the other hand, low skilled jobs were subject to rigorous monitoring and control and intensification of work pace. Moreover, there is an overall tendency to longer and flexible working hours.

As a result, many organizations have become more and more exclusive. They are designed and managed in a way that they are only accessible for people *without* disabilities and *with* the right qualifications, and only as long as they do meet the demands of the regular jobs. A growing number of people is not able to meet, or to keep up with these demands. They are referred to as “people with a distance to the labor market” or “vulnerable labor-market groups” (19). People with disabilities and/or no actual or currently requested professional qualification are part of this large and heterogeneous group that is growing in many countries (20).

## A Strategy for Changing the Organization of Work: IWR in a Nutshell

IWR is a strategy for changing the organization of work. Participative work redesign which implies active involvement of current employees, results in a redistribution of tasks in such a manner that job seekers with disabilities and/or lacking qualifications can support professionals in performing their key tasks, thereby contributing to the effective and efficient operation of staff as a whole. Both the resulting new jobs and the redefined jobs of incumbent staff should meet quality standards that guarantee health, safety and wellbeing.

The business logic behind this strategy is capitalizing on the widespread underutilization of skilled staff, who are a substantial amount of their working time engaged in tasks that do not need their qualification. Previous cost-saving reductions of “headcount” were often achieved by layoffs of lower qualified staff. The routine tasks they used to perform where at -least partially- transferred to the remaining higher qualified staff. For instance, in the Netherlands yearly nation wide surveys during the last decade reveal that one third of respondents are performing tasks below their qualification level (21). This phenomenon was confirmed in many IWR-consults by UWV^2^-professionals in such diverse branches as industry (wood, metal, leather, chemical, machine, bakery, meat processing, packaging), technical installation sector, temporary employment agencies, cleaning companies, waste disposal, whole sale business (vegetables, fruit, flowers), retail (department stores, supermarkets, distribution centers), hospitality (hotels, restaurants, catering), recreation (swimming baths, sports halls, amusement parks), health care (general hospital, mental hospital, home for the eldery, nursing home, sheltered home), public services (municipalities, counties, state), education (primary, secondary, higher, university). Despite labor shortage and unfilled vacancies, there is a substantial proportion of the population of working age that is still *not* engaged in work: *people with disabilities and/or mismatching skills*. However, given the proper conditions, work can be organized in such a way that these people can also make valuable contributions to any work organization. IWR originally was developed for people with disabilities and with little education, to organize assistant jobs based on elementary tasks. Later, the scope was broadened to all people with qualifications that have become deficient or even obsolete, or are at risk of becoming so: this gave rise to the development of IWR 2.0.^3^

IWR is a generic demand-oriented strategy for organizations to become more inclusive. The initial diagnostic phase consists of organizational analysis and inclusive work analysis. The organizational analysis results in a problem definition and a plan of action that embraces the challenges of the specific organization and that management is willing to adopt. If management believes the plan of action for redesign to be a possibly viable way to create additional staffing options, an inclusive work analysis of designated departments is performed. The results of this diagnostic phase are the input for participative work redesign. In collaboration with staff members and line management, redesign options, benefits and the preconditions for successful implementation will be discussed, preferably in work sessions. During these sessions, there is room to explore proposals of various kinds; they may concern redistribution of tasks to engage new colleagues with different qualifications and to make changes in existing jobs, as well as redefining roles and responsibilities, and alternative ways of onboarding, training, coaching, coordination and management. Based on these proposals, management has to establish the final setup of the redesign and the way it will be implemented.

### Organizational Analysis

Organizational analysis starts with exploring a company's or institution's mission and goals, its strategy to pursue these goals and the contextual factors the organization has to deal with (14). Focus of the analysis is on identifying existing or potential future bottlenecks in staffing vital to the organization's strategy.

Efforts necessary to guarantee sufficient qualified staff are dependent on changes in the external and internal labor market. Recently, despite pandemic related economic turmoil, many organizations in The Netherlands, Germany and several other countries are experiencing difficulties in attracting and retaining qualified staff due to tight labor market conditions. The shortage of qualified labor complicates recruitment of new staff and facilitates “job hopping” of current staff. In addition, organizations have to cope with threats to the employability of current staff. One important issue is advancing quality of work using countermeasures to the known work-related threats to the resilience of an aging population of workers facing higher retirement age. Organizations also need to keep their staff's skills and knowledge up to date and in line with innovations of products and services and keep their staff motivated and happy to increase retention. Most organizations are confronted with several of these challenges and have already tried to deal with them in their own way. It is important to connect to these ongoing activities and to explore if and how IWR can be of additional value. If IWR may be helpful and management is prepared to adapt work processes and change existing task distribution and work context, then the next step is inclusive work analysis.

#### Work Analysis

Organizational analysis yielded the scope for work analysis, the units that will be involved in this phase, and the specific personnel issues that need to be addressed. First, a clear picture of the present situation (“ist-situation”) is indispensable. It is needed in order to explore the opportunities for reallocating tasks to less qualified new staff, so that work pressure for current staff may be alleviated, or to create chances for their career development or the introduction of technological innovations. Traditional job analysis concerns critical tasks and responsibilities (roles) of a job holder, and the critical KSAs needed (22). Usually, this does not yield a complete inventory of the actual daily activities of the job holder, as that only becomes obvious in the analysis of work processes, as also performed in IWR.

Document study, observations and interviews are performed to identify the work processes, and the activities (tasks) of which they consist. For each task, frequency and duration are assessed, as well as who is (are) performing the task, what qualification is demanded, and if it is possible and desirable to transfer this task to someone less qualified. Finally, the conditions for transferring tasks are specified (“soll-situation”) in participative work redesign sessions.

## Case: Nursing Homes

Persisting shortage of staff in nursing homes is undermining both quality of work and quality of care. In the labor market, there are not nearly enough professionals who qualify for the great number of vacancies. Turnover is high, due to work pressure and lacking prospect for improvement, putting the burden on the remaining staff, including a substantial part of senior workers (over 55 years). There is not enough time for necessary professional development to deal with growing complexity in the demand for care, and for the introduction of new assistive technology.

This self-perpetuating spiral induces nursing homes to reconsider their traditional job structure and its relation to the actual demands in work processes. During the past decade, IWR has been successfully applied in general hospital settings to redistribute tasks and compose supporting jobs to make better use of professional staff capacity. Recently, IWR was applied in the setting of nursing homes to explore opportunities for organizational innovations that could contribute to the solution of the staffing problems.

In psychogeriatric units of a nursing home, inclusive work analyses yielded 72 different tasks, varying in size, that do not require a healthcare qualification at all. Nevertheless, professional nurses and caretakers spent some 245 h per week carrying out these duties, amounting to 30 percent of their working time. These tasks were part of 4 work processes: care, nutrition, wellbeing and domestic work. Redistribution of tasks in participative work redesign sessions with representatives of unit staff resulted in the definition of 3 new support functions (Table 1) in order to provide professional nurses and caretakers with the opportunity to focus on their core tasks.

-The “General assistant” role: assisting in keeping general residents areas clean and hygienic, escorting clients, and transport activities.
-The “Living assistant” role: paying attention to residents wellbeing, supporting and encouraging residents drink and food intake and supporting residents activities.
-The “Domestic assistant” role: ensuring the cleanliness and hygiene of the personal livings spaces of residents and the department in general.

**Table 1:** Three new assistant functions, examples of tasks.

**Work processes**	**General assistant**	**Living assistant**	**Domestic assistant**
Care	Escorting or transporting clients to and from living room and organization's restaurant	Supporting and encouraging residents' drink and food intake	Making beds, cleaning beds, collecting and transporting dirty laundry
Nutrition	Setting and clearing the tables Washing dishes	Preparing breakfast, serving coffee and tea	Cleaning living room and kitchens
Wellbeing	Socializing with clients	Activities with individual clients, e.g. reading, playing games, listening to music, taking a walk, supporting clients in the use of technical devices like “Braintrainer” or “ExperienceTV”	Socializing with clients
Domestic work	Daily cleaning of toilets, bathrooms, halls and corridors, doors and door handles Thorough cleaning of wheelchairs and rollators, hoists	Mopping floors of common areas like living rooms and kitchens	Vacuuming and mopping of clients' personal living spaces according to a fixed schedule

*Our organizations are cooperating in a transnational project on inclusive redesign, supported by the European Social Fund (project 2019EUSF2013)*.

The general assistant serves as entry job for new employees with low level KSAs. With sufficient training on the job, they can progress to one of the other assistant functions.

## IWR in Germany

In 2020 we started a project for a “proof of concept” of the inclusive redesign method in Germany. We wanted to learn if and how this approach could be applied in the German context of work organizations, as well as the social security system (23). The 10 students involved in this project were professionals employed by organizations of social security in Germany. They participated in the project as part of their course in work and organizational psychology at the HGU, the University of Applied Sciences of the German Social Accident Insurance (DGUV).

After receiving an introduction in theory and method of inclusive redesign, they started developing their skills at the campus by performing an inclusive work analysis of the university's restaurant staff. Next, they performed a redesign project at a local administrative agency of the DGUV. They performed an inclusive work analysis by observing and interviewing 30 staff members, and developed a proposal for redesign. In short: the redesign is aimed at redistributing tasks in such a way that at each level, functions are concentrated around their core duties, and that it creates job openings for people with disabilities and/or no or (currently) irrelevant professional qualifications. Unfortunately, the final phase of a participative redesign session together with the staff could not be realized, due to the start of the pandemic. The project report of the “ist-situation” describes the actual state of work processes, tasks and responsibilities, as well as staff's perceptions of workload, bottlenecks, ambitions and desirable changes in work and work context. For the “soll-situation”, a series of recommendations has been formulated.

The concept of inclusive redesign proved appealing to all involved: students, management and staff. The method proved to be well applicable in the context of a German work organization, and yielded useful results and recommendations, according to this organization's management. The results and the experiences in this feasibility study were encouraging for the next step ahead: a pilot in a German hospital struggling with staff shortages, especially of nurses. This pilot will start as soon as the Covid-situation allows.

## Discussion

In the Netherlands and Germany, but also many other countries growing demographic imbalance is responsible for structural labor market shortages and a structural more vulnerable aging workforce. Work organizations are challenged to develop new strategies in response to the resulting quantitative and qualitative staffing problems. One of the obvious directions to look for solutions are the large numbers of still unemployed people with disabilities and/or with qualifications not (longer) matching regular job vacancies. However, these job seekers are dependent on suitable jobs that enable (re)entry in organizations, and chances for professional development, that most organizations do not yet offer. To be able to make productive use of this growing labor market reserve and thus to create also better working conditions for current staff, a definite change in the way work is organized is necessary.

In the context of Social Security in The Netherlands IWR during the past decade has proven to be an effective instrument in demand-oriented service for employers, who want to include people with “a distance to the labor market”, in particular persons with disabilities^4^ Recently we have explored the applicability of IWR within the context of German Social Security and work organizations. The results and the experiences in this feasibility study were encouraging for the next step ahead: a pilot in a German hospital struggling with staff shortages, especially of nurses.

Inclusive work redesign (IWR) is a change strategy for organizations interested in an attractive business solution: the combined use of internal and external untapped human resources. Recent publications on human resource management (HRM) are calling for “Inclusive HRM” (24) and “Employer Engagement” (19) as “the active involvement of employers in addressing the societal challenge of promoting the labor-market participation of vulnerable groups”. Inclusive work redesign as a generic participative change strategy can be a valuable asset in pursuing these goals.

## Data Availability Statement

Publicly available case studies used or this study will be accessible on request.

## Author Contributions

All authors listed have made a substantial, direct, and intellectual contribution to the work and approved it for publication.

## Conflict of Interest

The authors declare that the research was conducted in the absence of any commercial or financial relationships that could be construed as a potential conflict of interest.

## Publisher's Note

All claims expressed in this article are solely those of the authors and do not necessarily represent those of their affiliated organizations, or those of the publisher, the editors and the reviewers. Any product that may be evaluated in this article, or claim that may be made by its manufacturer, is not guaranteed or endorsed by the publisher.

## Footnotes

^1^ https://www.youtube.com/watch?v=gXnEnWZ5MX8&t=8s

^2^UWV, the Dutch Employee Insurance Agency.

^3^ https://www.inclusiveworkredesign.com/methods-instruments/inclusive-redesign-of-work-20

^4^ https://www.inclusiveworkredesign.com
